# Supplementary material for: HIVprotI: an integrated web based platform for prediction and design of HIV proteins inhibitors
Source: J Cheminform. 2018 Mar 9;10:12. doi: 10.1186/s13321-018-0266-y (PMC5845081; doi:10.1186/s13321-018-0266-y)
Supplement: Supplementary file 2 — Additional file 2. Source code of HIVProtI web server. [file 13321_2018_266_MOESM2_ESM.zip › HIVprotI_Source-code/hivprotI/weka-3-6-6/doc/weka/gui/GenericPropertiesCreator.html]

GenericPropertiesCreator


---


|  |  |  |
| --- | --- | --- |
| **Overview** \| **Package** \| **Class** \| **Tree** \| **Deprecated** \| **Index** \| **Help** \| **Weka's home** | |  |
| **PREV CLASS**   **NEXT CLASS** | **FRAMES**    **NO FRAMES**     **All Classes** |  |
| SUMMARY: NESTED \| FIELD \| CONSTR \| METHOD | DETAIL: FIELD \| CONSTR \| METHOD |  |


---


## weka.gui Class GenericPropertiesCreator

```
java.lang.Object
  weka.gui.GenericPropertiesCreator
```

---

``` public class GenericPropertiesCreator extends java.lang.Object ```

This class can generate the properties object that is normally loaded from
the `GenericObjectEditor.props` file (= PROPERTY\_FILE). It takes
the `GenericPropertiesCreator.props` file as a template to
determine all the derived classes by checking the classes in the given
packages (a file with the same name in your home directory overrides the
the one in the weka/gui directory/package).   
E.g. if we want to have all the subclasses of the `Classifier`
class then we specify the superclass ("weka.classifiers.Classifier") and the
packages where to look for ("weka.classifiers.bayes" etc.):

```
   weka.classifiers.Classifier=\
     weka.classifiers.bayes,\
     weka.classifiers.functions,\
     weka.classifiers.lazy,\
     weka.classifiers.meta,\
     weka.classifiers.trees,\
     weka.classifiers.rules
```

This creates the same list as stored in the
`GenericObjectEditor.props` file, but it will also add
additional classes, that are not listed in the static list (e.g. a newly
developed Classifier), but still in the classpath.   
  
For discovering the subclasses the whole classpath is inspected, which means
that you can have several parallel directories with the same package
structure (e.g. a release directory and a developer directory with additional
classes).   
  
The dynamic discovery can be turned off via the `UseDyanmic`
property in the props file (values: true|false).

**Version:**
:   $Revision: 7059 $

**Author:**
:   FracPete (fracpete at waikato dot ac dot nz)

**See Also:**: `CREATOR_FILE`, `PROPERTY_FILE`, `USE_DYNAMIC`, `GenericObjectEditor`, `ClassDiscovery`

---

| **Field Summary** | |
| --- | --- |
| `static java.lang.String` | `USE_DYNAMIC`             name of property whether to use the dynamic approach or the old GenericObjectEditor.props file |
| `static boolean` | `VERBOSE`             whether to output some debug information |


**Constructor Summary**

`GenericPropertiesCreator()`
  
          initializes the creator, locates the props file with the Utils class.

`GenericPropertiesCreator(java.lang.String filename)`
  
          initializes the creator, the given file overrides the props-file search
of the Utils class


| **Method Summary** | |
| --- | --- |
| `void` | `execute()`             generates the props-file for the GenericObjectEditor and stores it |
| `void` | `execute(boolean store)`             generates the props-file for the GenericObjectEditor and stores it only if the the param `store` is TRUE. |
| `boolean` | `getExplicitPropsFile()`             returns TRUE, if a file is loaded and not the Utils class used for locating the props file. |
| `java.lang.String` | `getInputFilename()`             returns the name of the input file |
| `java.util.Properties` | `getInputProperties()`             returns the input properties object (template containing the packages) |
| `java.lang.String` | `getOutputFilename()`             returns the name of the output file |
| `java.util.Properties` | `getOutputProperties()`             returns the output properties object (structure like the template, but filled with classes instead of packages) |
| `static void` | `main(java.lang.String[] args)`             for generating props file: no parameter: see default constructor 1 parameter (i.e., filename): see default constructor + setOutputFilename(String) 2 parameters (i.e, filenames): see constructor with String argument + setOutputFilename(String) |
| `void` | `setExplicitPropsFile(boolean value)`             if FALSE, the locating of a props-file of the Utils-class is used, otherwise it's tried to load the specified file |
| `void` | `setInputFilename(java.lang.String filename)`             sets the file to get the information about the packages from. |
| `void` | `setOutputFilename(java.lang.String filename)`             sets the file to output the properties for the GEO to |
| `boolean` | `useDynamic()`             gets whether the dynamic approach should be used or not |

**Methods inherited from class java.lang.Object**

`equals, getClass, hashCode, notify, notifyAll, toString, wait, wait, wait`

**Field Detail**

### VERBOSE

```
public static final boolean VERBOSE
```

:   whether to output some debug information

    **See Also:**: Constant Field Values

---


### USE\_DYNAMIC

```
public static final java.lang.String USE_DYNAMIC
```

:   name of property whether to use the dynamic approach or the old
    GenericObjectEditor.props file

    **See Also:**: Constant Field Values


**Constructor Detail**

### GenericPropertiesCreator

```
public GenericPropertiesCreator()
                         throws java.lang.Exception
```

:   initializes the creator, locates the props file with the Utils class.

    **Throws:**: `java.lang.Exception` - if loading of CREATOR\_FILE fails **See Also:**: `CREATOR_FILE`, `Utils.readProperties(String)`, `loadInputProperties()`

---


### GenericPropertiesCreator

```
public GenericPropertiesCreator(java.lang.String filename)
                         throws java.lang.Exception
```

:   initializes the creator, the given file overrides the props-file search
    of the Utils class

    **Parameters:**: `filename` - the file containing the packages to create a props file from **Throws:**: `java.lang.Exception` - if loading of the file fails **See Also:**: `CREATOR_FILE`, `Utils.readProperties(String)`, `loadInputProperties()`


**Method Detail**

### setExplicitPropsFile

```
public void setExplicitPropsFile(boolean value)
```

:   if FALSE, the locating of a props-file of the Utils-class is used,
    otherwise it's tried to load the specified file

    :   **Parameters:**: `value` - if true the specified file will be loaded not via the Utils-class **See Also:**: `Utils.readProperties(String)`, `loadInputProperties()`

---


### getExplicitPropsFile

```
public boolean getExplicitPropsFile()
```

:   returns TRUE, if a file is loaded and not the Utils class used for
    locating the props file.

    :   **Returns:**: true if the specified file is used and not the one found by the Utils class **See Also:**: `Utils.readProperties(String)`, `loadInputProperties()`

---


### getOutputFilename

```
public java.lang.String getOutputFilename()
```

:   returns the name of the output file

    :   **Returns:**: the name of the output file

---


### setOutputFilename

```
public void setOutputFilename(java.lang.String filename)
```

:   sets the file to output the properties for the GEO to

    :   **Parameters:**: `filename` - the filename for the output

---


### getInputFilename

```
public java.lang.String getInputFilename()
```

:   returns the name of the input file

    :   **Returns:**: the name of the input file

---


### setInputFilename

```
public void setInputFilename(java.lang.String filename)
```

:   sets the file to get the information about the packages from.
    automatically sets explicitPropsFile to TRUE.

    :   **Parameters:**: `filename` - the filename for the input **See Also:**: `setExplicitPropsFile(boolean)`

---


### getInputProperties

```
public java.util.Properties getInputProperties()
```

:   returns the input properties object (template containing the packages)

    :   **Returns:**: the input properties (the template)

---


### getOutputProperties

```
public java.util.Properties getOutputProperties()
```

:   returns the output properties object (structure like the template, but
    filled with classes instead of packages)

    :   **Returns:**: the output properties (filled with classes)

---


### useDynamic

```
public boolean useDynamic()
```

:   gets whether the dynamic approach should be used or not

    :   **Returns:**: true if the dynamic approach is to be used

---


### execute

```
public void execute()
             throws java.lang.Exception
```

:   generates the props-file for the GenericObjectEditor and stores it

    :   **Throws:**: `java.lang.Exception` - if something goes wrong **See Also:**: `execute(boolean)`

---


### execute

```
public void execute(boolean store)
             throws java.lang.Exception
```

:   generates the props-file for the GenericObjectEditor and stores it only
    if the the param `store` is TRUE. If it is FALSE then the
    generated properties file can be retrieved via the `getOutputProperties`
    method.

    :   **Parameters:**: `store` - if TRUE then the properties file is stored to the stored filename **Throws:**: `java.lang.Exception` - if something goes wrong **See Also:**: `getOutputFilename()`, `setOutputFilename(String)`, `getOutputProperties()`

---


### main

```
public static void main(java.lang.String[] args)
                 throws java.lang.Exception
```

:   for generating props file:

    - no parameter:
      see default constructor
    - 1 parameter (i.e., filename):
      see default constructor + setOutputFilename(String)
    - 2 parameters (i.e, filenames):
      see constructor with String argument + setOutputFilename(String)

    :   **Parameters:**: `args` - the commandline arguments **Throws:**: `java.lang.Exception` - if something goes wrong **See Also:**: `GenericPropertiesCreator()`, `GenericPropertiesCreator(String)`, `setOutputFilename(String)`


---


|  |  |  |
| --- | --- | --- |
| **Overview** \| **Package** \| **Class** \| **Tree** \| **Deprecated** \| **Index** \| **Help** \| **Weka's home** | |  |
| **PREV CLASS**   **NEXT CLASS** | **FRAMES**    **NO FRAMES**     **All Classes** |  |
| SUMMARY: NESTED \| FIELD \| CONSTR \| METHOD | DETAIL: FIELD \| CONSTR \| METHOD |  |


---
